# Effects of Internet Use on Health and Depression: A Longitudinal Study

**DOI:** 10.2196/jmir.1149

**Published:** 2010-03-12

**Authors:** Katie Bessière, Sarah Pressman, Sara Kiesler, Robert Kraut

**Affiliations:** ^2^Department of PsychologyUniversity of KansasLawrenceUSA; ^1^Human-Computer Interaction InstituteCarnegie Mellon UniversityPittsburghUSA

**Keywords:** Depression, health, social support, Internet, longitudinal survey

## Abstract

**Background:**

The rapid expansion of the Internet has increased the ease with which the public can obtain medical information. Most research on the utility of the Internet for health purposes has evaluated the quality of the information itself or examined its impact on clinical populations. Little is known about the consequences of its use by the general population.

**Objective:**

Is use of the Internet by the general population for health purposes associated with a subsequent change in psychological well-being and health? Are the effects different for healthy versus ill individuals? Does the impact of using the Internet for health purposes differ from the impact of other types of Internet use?

**Methods:**

Data come from a national US panel survey of 740 individuals conducted from 2000 to 2002. Across three surveys, respondents described their use of the Internet for different purposes, indicated whether they had any of 13 serious illnesses (or were taking care of someone with a serious illness), and reported their depression. In the initial and final surveys they also reported on their physical health. Lagged dependent variable regression analysis was used to predict changes in depression and general health reported on a later survey from frequency of different types of Internet use at an earlier period, holding constant prior depression and general health, respectively. Statistical interactions tested whether uses of the Internet predicted depression and general health differently for people who initially differed on their general health, chronic illness, and caregiver status.

**Results:**

Health-related Internet use was associated with small but reliable increases in depression (ie, increasing use of the Internet for health purposes from 3 to 5 days per week to once a day was associated with .11 standard deviations more symptoms of depression, *P* = .002). In contrast, using the Internet for communication with friends and family was associated with small but reliable decreases in depression (ie, increasing use of the Internet for communication with friends and family purposes from 3 to 5 days per week to once a day was associated with .07 standard deviations fewer symptoms of depression, *P* = .007). There were no significant effects of respondents’ initial health status (*P* = .234) or role as a caregiver (*P* = .911) on the association between health-related Internet use and depression. Neither type of use was associated with changes in general health (*P* = .705 for social uses and *P* = .494 for health uses).

**Conclusions:**

Using the Internet for health purposes was associated with increased depression. The increase may be due to increased rumination, unnecessary alarm, or over-attention to health problems. Additionally, those with unmeasured problems or those more prone to health anxiety may self-select online health resources. In contrast, using the Internet to communicate with friends and family was associated with declines in depression. This finding is comparable to other studies showing that social support is beneficial for well-being and lends support to the idea that the Internet is a way to strengthen and maintain social ties.

## Introduction

The rapid expansion of the Internet has greatly increased the amount of health information available to the general public. In 2001, approximately 43% of the US public was using the Internet, and an estimated 40% reported using Internet health resources in the previous year [1]. More recently, in 2009, about 74% [2] of US adults said they used the Internet. While email and general information searches remain the most common uses of the Internet [3], using the Internet to obtain health resources is also very popular [4], with over 50% of Internet users searching for medical information [2]. With millions using the Internet, and a large proportion of the population explicitly using it for health purposes, it is important to assess how this particular use of the Internet is affecting people's well-being, especially their physical and mental health. Although prior research has shown that use of the Internet to communicate with friends and family is associated with declines in depression [5], little reliable information exists about the impact of using the Internet to obtain health resources, especially in nonclinical populations [6-8]. The current study used data from a national US random household sample survey to address the impact of Internet use to obtain health information and support on well-being and health. We also examined whether these effects differed for people with differing levels of health and caregiver status, and whether these well-being associations were present for other types of Internet use.

### Health Resources Online

Traditionally, physicians held and filtered health information for the general public [9]. Given the easy availability of health information online, people can now bypass medical professionals entirely and find information, advice, support, and even treatments (eg, pharmaceuticals and herbal remedies) on the Internet [6]. The Internet contains thousands of medically relevant websites such as large information sites (eg, WebMD and Medline), disease support groups, discussion boards, distributions lists, personal websites, and websites selling “miracle cures”. Online communities and support groups allow people to talk about their health problems with others and are popular both as a source of social support and as a primary source of information [10-12]. Online communities and support groups also provide support to caregivers and the families of people with serious health problems [13].

Much of the research examining the effects of these online health resources has examined its use by groups with a specific illness or disease [14]. Although some writers have warned about poor quality medical information online [8] and patients’ inability to distinguish poor advice from good advice [10], there are few reports of serious harm [15]. Both qualitative studies [16] and polls assessing consumers’ beliefs [13,17] suggest that online health information and health support can improve patients’ and caregivers’ optimism and feelings of control. For example, 32% of respondents who used one consumer health site reported their condition had improved after visiting the site, 64% said they felt they were better informed, and 50% said they believed that the information they learned changed how they felt about their condition [18]. These types of retrospective data are unreliable on many dimensions, including that typically do not compare Internet users’ reports of health benefits to those of non-users. However, they are one source for the popular belief that use of the Internet for health purposes can improve well-being. These self-report studies are supported by clinical trials of online interventions, which suggest that these programs can reduce patients’ pain and stress and improve their physical performance and perceived social support [7,19,20]. Many studies of online health resources include the caregivers of the chronically ill in the sample [21] because of the high levels of stress experienced by this population as well as their strong interest in the health of those they care for [22]. Overall, little is known about the extent to which individuals with health problems (or caregivers of the ill) benefit from having unfettered access to online health information and support. The literature cited above would lead one to believe that online health information might be beneficial to patients and could certainly lead people to feel more in control of their interactions with medical providers, reassure them about ordinary aches and pains, increase their understanding of others’ health problems, and improve their preventive health care and physical condition.

Although having a serious illness is one factor that leads people to use the Internet for medical purposes [23], there are also millions of healthy individuals who use online health resources [24,25]. About three-quarters of visitors to one health website had no particular health condition or illness, and half of searches concerned another person [18]. These healthy individuals may be seeking information about a friend’s illness, or they may be educating themselves about diseases in the news. They may also be seeking information about their own minor or ambiguous symptoms or looking into other health-relevant concerns such as baldness or weight loss. While this type of Internet use in healthy individuals seems harmless, these individuals may receive unnecessary or alarming information about minor problems [26] and may focus too much attention on disease processes and symptoms, leading them to ruminate about their health [27]. Rumination increases pessimism [28] and increases depression symptoms [29]. Reading about disease might increase people’s health anxiety, reinforce hypochondriasis, or cause them unnecessary concern about the health status of themselves or loved ones [30]. Online health websites might even lead people to purchase harmful drugs or engage in risky health practices. Given some poor quality health information available online and the possibility that reading about health online may induce unnecessary health-related concerns, the use of online health information by healthy people may harm their psychological well-being.

### Nonmedical Internet Use

In addition to providing health-related information and access to online health support groups, the Internet may influence health and well-being by influencing the ease with which people can access social support from family and friends. Social communication with friends and family is arguably the most important use of the Internet [31]. For example, on a typical day Americans are more likely to use the Internet to send and receive email than for any other type of Internet use (eg, search engines, news, medical and weather) [4]. Researchers have argued that communication on the Internet augments social resources by providing an added avenue for interaction, that is, a way to keep up with distant friends and family, and that this could lead to a larger social network [32-37]. The implication is that those who use the Internet to communicate with friends and family will show well-being benefits, which is consistent with work showing that communication (and the ensuing social resources) is associated with better psychological functioning, lower stress, and greater positive affect [38,39]. By contrast, those who communicate little and have fewer social resources have poorer psychological functioning and higher levels of depression [40,41]. While some research has suggested that Internet communication is also associated with better psychological well-being [42], interacting with strangers online may harm psychological well-being, especially among those who have already-existing social support and other social resources [43], perhaps by displacing strong social ties with weaker ones.

Excessive use of the Internet for gaming and gambling has been tied to increased depression [44,45], as has use of the Internet for shopping [46]. While suggestive of possible negative outcomes from using the Internet for entertainment, these studies have primarily been done as case studies of addicted individuals and/or as evidence for Internet pathology. It is not clear whether less excessive amounts of Internet use for these purposes would have similar negative outcomes. Finally, use of the Internet for escape may have both positive and negative outcomes, resulting in the overall impact on well-being remaining relatively neutral. Going online to escape and relax has been shown to be a source of gratification to Internet users and is a predictor of heavier Internet usage [47]. This may have positive well-being outcomes since using the Internet to escape and relax may be viewed as a coping strategy [48] or as a restorative activity [49] that helps people “recharge their batteries.” On the other hand, individuals likely to say that they use the Internet for escapism are often doing so to alleviate feelings of depression and isolation [50] or to divert themselves from something stressful and negative going on in the offline world (eg, HIV treatment) [51]. Again, this may be beneficial as a coping strategy and/or relaxation technique, but if this escapism occurs at pathological levels it may also lead to harmed social relationships, negative health consequences (from a lack of activity), and further depression.

### Current Study

The purpose of the current research was to determine whether using the Internet for health purposes is beneficial or harmful to physical and psychological well-being. We were also interested in whether this association would be moderated by people's health or care-giving status. Specifically, we examined whether individuals with a good reason for searching the Internet for medical information would fare differently from their healthy (or non care-giving) counterparts. We were also interested in whether the impact on well-being of using the Internet for medical purposes could be isolated from the effects of other types of Internet use (ie, is harm to well-being simply a function of time spent online?). Specifically, we examined the impact of Internet use in six domains—communication with friends and family, meeting new people, news and other information access, entertainment and escape, shopping, and health—on Internet users’ depression and general health from 6 to 18 months later. We anticipated that using the Internet for health purposes would lead to better health and less depression among participants in poor health and in their caregivers, but that the direction of these effects would be the opposite among healthy people. To test these hypotheses, we studied the impact of three moderating factors: participants’ initial general health, whether or not they currently had a serious illness, and whether or not they were the caregivers of someone with a serious illness. We also hypothesized that using the Internet for communication with friends and family would be associated with well-being benefits, consistent with previous work showing the positive effects of social support and relationships. We did not predict that using the Internet in other ways would have any impact on well-being.

## Methods

### Procedure and Participants

In 2000, we conducted a national sample survey of US households using random digit dialing and a panel design with three questionnaires delivered over an 18-month period. Those answering the phone were asked to list members of their household and specify if they had Internet access; all persons 19 years and older were solicited for the survey. We then sent surveys to all 2700 respondents who agreed to participate. Because of our interest in the consequences of Internet use, we oversampled on Internet access so that approximately 75% of those surveyed came from Internet-enabled households at a time when about 50% of US household had Internet access [52]. To encourage responses, participants had a choice of taking the survey in paper or electronic form [53]. Prior research has shown that electronic and paper versions of standard survey instruments are comparable [54]. We sent potential respondents a personalized pre notice, a cover letter, a consent form, a US $10 honorarium, a pointer to an electronic survey if they had Internet access, a paper survey if they had no Internet access or preferred paper, and up to three personalized follow-up reminders [55]. We also mailed a paper survey to any participant with an invalid email address and to those requiring a third reminder. Sixty percent of the participants completed the surveys online. Participants completed their first survey from June to September 2000, and their final survey 12 to 18 months later.

Forty-five percent of this sample, or 1222 adults, completed the first survey; of these, 74% reported having Internet access. Approximately six months after they responded, we sent those who completed the first survey a follow-up survey, which 82.8%, or 1011 people, completed (37% of the original sample). These 1222 respondents were sent a third survey approximately 6 months later. Of the 1222 people who completed the initial survey, 60.5%, or 740 people responded to the third survey (27% of the original sample).

Participants’ ages ranged from 13 to 101 (85% were 19 years or older), with a median age of 44 years. Forty-three percent were men, 89% were Caucasian, and 61% were married. Participants’ median household income was between US $30,000 and US $50,000. Thirty percent had a household income of US $30,000 or less; 44% had a household income between US $30,000 and US $70,000; 26% had a household income of US $70,000 or more. Compared with 2000 US Census data, our sample was older (median for sample = 44 years vs census median = 35.3 years), had fewer men (sample = 43% vs census = 49.1%), more Caucasians (sample = 89% vs census = 75.1%), and a higher median household income (sample = US $30-50,000 vs census = US $41,900). Internet users in this sample were younger and wealthier than nonusers, mirroring national trends among Internet users [3]. These differences in our sample compared with characteristics of the US population as a whole suggest caution in generalizing our findings to the population as a whole.

### Measures

#### Demographic Control Variables

Participants reported their gender (coded male = 1, female = 0), age, marital status (coded as married = 1, not married = 0), education level (coded from some elementary school = 1 to post graduate school = 7), race (coded as white = 1, other = 0), and household income per year (coded as less than US $10,000 = 1 to US $70,000 or more = 8).

#### Illness

Presence of a serious illness was measured by asking the participants whether they had any of the following health problems: heart disease or serious heart problem, liver disease (eg, cirrhosis), cancer, disabling arthritis, stroke, serious immune disease (eg, multiple sclerosis or lupus), long term injury from an accident, Alzheimer's or dementia, clinical depression or mental illness, alcohol or drug problem, disability or developmental disorder, lung problem (eg, emphysema or asthma), serious digestive system problems such as Crohn's disease, and nervous system or seizure disorder. Twenty-one percent of the sample completing the first questionnaire reported having at least one of these illnesses (coded as having at least one disease = 1, none = 0).

#### Caregiver Status

Participants indicated whether they were responsible for the care of anyone having any of the aforementioned illnesses (coded yes = 1, no = 0). Sixteen percent of participants in the first questionnaire reporting taking care of someone with a serious illness.

#### Depression

Depression was measured using a 12-item version of the Center for Epidemiologic Studies of Depression Scale (CES-D) [56]. The CES-D was designed to measure current levels of depressive symptomatology, including depressed mood, feelings of guilt and worthlessness, feelings of helplessness and hopelessness, loss of appetite, sleep disturbance, and psychomotor retardation. The scale measures the frequency of symptoms of depression among healthy, nondepressed populations as well as among those with clinical disorders. Although the scale can reliably distinguish clinically depressed individuals from those who are not depressed as diagnosed by interview measures like the structured clinical interview for the Diagnostic and Statistical Manual of Mental Disorders, Fourth Edition (DSM-IV) [57], it is more frequently used to assess the degree of depression in the general population. We used the CES-D as a continuous measure of degree of depression rather than using a cut-off to classify participants as clinically depressed for two reasons. First, prior research indicates that those with higher levels of depression face worse health outcomes even if they are not clinically depressed [58,59]. Second, a continuous measure of depression is more sensitive than a dichotomous one, thereby reducing the type II error of failing to identify relationships that actually exist. Respondents described how frequently they experienced various symptoms of depression in the past week, with 1 = no days with a symptom, and 4 = experienced the symptom 5 to 7 days in the preceding week (Cronbach alpha = .89).

#### General Health

We asked the one-item general health question from the 36-item Medical Outcome Study Short-Form Health Survey (MOS SF-36) [60,61]. The item asked, “In general, would you say your health is…” scored from 1 = poor to 5 = excellent. Because of an error, this item was omitted from the second survey and measured only on the first and third ones.

#### Internet Uses

Respondents described how often they had used the Internet for 27 different purposes in the previous six months. They made these estimates on a 7-point, logarithmic-like scale, in which a unit increase represented an approximate doubling of Internet use. The choices were: “never,” “less often” (than every few weeks), “every few weeks,” “1-2 days per week,” “3-5 days per week,” “about once a day,” and “several times a day.” We grouped these 27 items into six scales based on prior exploratory factor analyses from an independent sample of 446 respondents. The six scales were: communicating with friends and family, communicating in online groups and to meet people, retrieving and using non-health information, retrieving and using health information, seeking entertainment or escape, and shopping [37].

We conducted a confirmatory factor analysis in the current sample to test whether the six-factor model better explained the data than a single-factor model. The single-factor model represented the hypothesis that Internet use is best measured by a single index. The single-factor model was a poor fit to the data (comparative fit index [CFI] = .81), whereas a six-factor model was a significantly better fit (CFI = .90). Listed below are the Internet-use scales examined in this research and the Chronbach alpha measure of internal reliability of the items that make up each scale.

1. Communicating with family and friends included items that measured “communicating with someone in your local area,” “keeping in touch with someone far away,” “communicating with friends,” and “communicating with relatives.” (Cronbach alpha = .95).
2. Communicating to meet new people and/or in online groups included items that measured “meeting new people for social purposes,” and “participating in an online group.” (Cronbach alpha = .81).
3. Informational uses included items that measured “getting news online,” “finding information about local events,” “finding information about national or international events,” “finding information about movies, books, or other leisure activities,” “getting information for work or school,” and “getting information for a hobby.” (Cronbach alpha = .94).
4. Entertainment/escape included items that measured “killing time,” “releasing tension,” “overcoming loneliness,” “being entertained,” “playing games,” and “listening to music.” (Cronbach alpha = .94).
5. Shopping included items that measured “buying products or services,” “getting information about products,” and “making a travel reservation.” (Cronbach alpha = .65).
6. Health included items that measured “getting information about a health concern or medical problem,” “getting information about ways to prevent illness,” and “communicating with others about health concerns or problems.” (Cronbach alpha = .84).

### Statistical Analysis

To test hypotheses about changes in participants’ general health and levels of depression, we used regression analysis with a lagged dependent variable, as recommended by Cohen et al [62]. This analysis predicts participants’ levels of general health or depression at a later time period from variables measured at an earlier time period, including control variables, measures of Internet use, and the lagged dependent variable (ie, their prior health or depression score). Since the health outcome was assessed only on the first questionnaire (at time 1) and on the third questionnaire (at time 3), there is only a single lagged dependent variable and thus a single observation per respondent; changes in general heath, therefore, were analyzed using ordinary least-squares regression. However, depression was assessed on all three questionnaires (at times 1, 2, and 3). Therefore, there are two lagged dependent variables measuring depression (depression at time 2 controlling for depression at time 1 and depression at time 3 controlling for depression at time 2). This resulted in two observations per respondent. To adjust both the coefficients and standard errors for the nonindependence of observations within respondents, we analyzed changes in depression using random effects regression, with the respondent as the random effect.

Because prior levels of the outcome variables (ie, health and depression) are included in the analyses, both the dependent variables and the other predictor variables have been adjusted for the initial levels of health or depression. Lagged dependent variable regression is appropriate for testing dynamic theories of change, in which states or events at one time influence states or events at a later time [63]. Lagged dependent variable models are appropriate when the data exhibit “stationarity,” that is, the dependent variable at each time period comes from the same population with a common mean and variance, and the serial correlation in the residuals is not high (ie, less than .50). Preliminary tests indicated that these conditions were met, making lagged dependent variable regression appropriate for the research reported here.

The sample includes all respondents who had change scores on the depression and health outcomes: 916 respondents who completed the CES-D depression index at least twice and 671 who described their general health twice. We included in the analyses participants who had never used the Internet. Our analyses did not change when we eliminated respondents who had never used the Internet. Some respondents omitted one or more independent variables (such as their initial health status, income or estimates of Internet use), causing a reduction in sample size in the regressions and potential biases in results if the respondents with missing data were not a random subset of the full sample. To correct for these problems, we used multiple imputation to fill in missing values, as suggested by Rubin [64]. Multiple imputation creates multiple datasets that estimate missing independent variables from the nonmissing data. These data are then analyzed using conventional statistical methods and combined to produce estimates and confidence intervals that incorporate the uncertainty resulting from imputing missing data [65]. Our imputation model included all the analysis variables as well as respondents’ self-reports on the number of hours per week they used the Internet at home and at work for electronic mail and accessing the World Wide Web. We used the multiple imputation procedure in Stata version 11 (StataCorp LP, College Station, TX, USA) [66]. We then replicated the results using the multiple imputation (MI) procedure in SAS for Windows version 9.1 (SAS Institute Inc, Cary, NC, USA) [67], which assumes the data have an underlying multivariate normal distribution. The results were similar regardless of whether they involved multiple imputation or not and whether the imputation was based on the Stata or SAS procedures. To simplify the presentation, therefore, we present only the results based on the Stata multiple imputation procedure.

## Results

### Preliminary Analyses

Descriptive statistics for the sample and correlations among variables are presented in Multimedia Appendix 1. The correlations showed moderate associations among all the Internet use variables (mean *r* = .50). In addition, older respondents were less likely to use the Internet than younger ones (mean *r* between various Internet use measures and age = -.26). The outcome measures were relatively stable (correlation between general health at times 1 and 3, *r* = .69 and between depression at times 1 and 3, *r* = .59). The measures of general health and depression were themselves moderately negatively correlated (mean *r* at a single time period = -.35).

We conducted a preliminary regression analysis using demographic controls and measures of initial health status to predict the six different components of Internet use at time 1. When predicting use of the Internet for a specific purpose we included the other components of Internet use as predictor variables to control for overall propensity to use the Internet. These analyses are summarized in Tables 1 and 2. As shown in Models 1A to 1F, at the time of the first survey, women were more likely to use the Internet to communicate with friends and family online and for health purposes, whereas men were more likely to use the Internet for information and escape. Younger participants reported more use of the Internet to meet people and participate in online groups and for information and entertainment, whereas older participants reported more use of the Internet for health purposes. Married respondents were less likely than single respondents to use the Internet to communicate with friends and family and to meet new people. More highly educated respondents were more likely to use the Internet to communicate with friends and family, for information, and for shopping, but less likely to use it for entertainment. Wealthier respondents were more likely to use the Internet to communicate with friends and family, for information, and for shopping, but less likely to use it for health purposes. These demographic results echo results of national polls [68].

Of particular interest to the present research is that at the time of the first questionnaire, respondents who had a serious illness themselves or those who cared for someone with a serious illness were more likely to use the Internet for health purposes. Those who were more depressed at the time of the first questionnaire were more likely to use the Internet for escape and to obtain health resources, but were less likely to use it for communicating with friends and family or for shopping.

**Table 1:** Predicting different uses of the Internet at time 1 from demographic, health, and well-being variables (models 1A to 1C)

	Model 1A				Model 1B				Model 1C			
	Internet: Family & Friends				Internet: Meet People				Internet: Information			
Variable	Beta	SE	*t*	*P*	Beta	SE	*t*	*P*	Beta	SE	*t*	*P*
Intercept	-.09	.10	-.86	.39	.05	.10	.48	.63	-.24	.09	-2.52	.01
Male	-.33	.06	-5.49	.00	.07	.06	1.20	.23	.35	.05	6.83	.00
Age	-.05	.04	-1.13	.26	-.15	.04	-4.11	.00	-.17	.04	-4.67	.00
White	.25	.10	2.58	.01	-.03	.11	-.28	.78	-.04	.09	-.44	.66
Married	-.27	.07	-3.95	.00	-.12	.06	-1.90	.06	.00	.07	.07	.94
Education	.15	.03	4.50	.00	-.03	.04	-.83	.41	.14	.03	4.23	.00
Income	.15	.04	4.24	.00	-.02	.03	-.50	.62	.07	.03	2.55	.01
Depression	-.07	.03	-2.38	.02	.01	.03	.48	.63	.02	.03	.72	.47
Serious illness	.02	.03	.76	.45	.00	.03	-.08	.94	-.06	.03	-2.31	.02
Caregiver	-.03	.03	-1.22	.23	-.01	.03	-.44	.66	-.03	.02	-1.30	.20
General health	.07	.03	2.15	.03	.01	.03	.27	.79	.00	.03	.02	.99
Internet: family & friends					.18	.03	6.10	.00	.17	.03	5.84	.00
Internet: meet people	.16	.03	5.49	.00					.04	.03	1.34	.18
Internet: information	.21	.04	5.79	.00	.05	.04	1.37	.17				
Internet: entertainment	.28	.04	7.33	.00	.25	.04	6.53	.00	.19	.03	5.88	.00
Internet: shopping	.08	.04	2.35	.02	-.07	.03	-1.94	.05	.30	.03	9.38	.00
Internet: health	.05	.03	1.58	.11	.22	.03	7.33	.00	.18	.03	6.52	.00
												
R-squared	.29				.27				.45			

**Table 2:** Predicting different uses of the Internet at time 1 from demographic, health, and well-being variables (models 1D to 1F)

	Model 1D				Model 1E				Model 1F			
	Internet: Entertainment				Internet: Shopping				Internet: Health			
Variable	Beta	SE	*t*	*P*	Beta	SE	*t*	*P*	Beta	SE	*t*	*P*
Intercept	.12	.09	1.29	.20	-.17	.09	-1.81	.07	.19	.10	1.87	.06
Male	.18	.05	3.28	.00	.09	.05	1.66	.10	-.27	.06	-4.80	.00
Age	-.11	.03	-3.08	.00	-.01	.03	-.42	.68	.15	.03	4.38	.00
White	-.20	.08	-2.44	.02	.02	.09	.22	.82	-.05	.09	-.51	.61
Married	-.08	.06	-1.29	.20	.06	.06	.98	.33	.07	.06	1.06	.29
Education	-.21	.03	-7.08	.00	.15	.03	4.56	.00	.00	.03	.09	.93
Income	.02	.03	.57	.57	.08	.03	2.37	.02	-.09	.03	-2.83	.01
Depression	.15	.03	5.75	.00	-.08	.03	-2.65	.01	.07	.03	2.62	.01
Serious illness	.02	.03	.55	.59	-.01	.03	-.22	.83	.08	.03	3.06	.00
Caregiver	.02	.03	.77	.44	.05	.02	1.81	.07	.05	.02	2.02	.05
General health	-.05	.03	-1.55	.12	.01	.03	.30	.77	-.03	.03	-.94	.35
Internet: family & friends	.22	.03	7.05	.00	.07	.03	2.25	.03	.05	.03	1.65	.10
Internet: meet people	.18	.03	6.20	.00	-.05	.03	-1.83	.07	.20	.03	7.80	.00
Internet: information	.19	.03	5.70	.00	.34	.04	9.80	.00	.22	.03	6.44	.00
Internet: entertainment					.17	.04	4.61	.00	.02	.03	.58	.57
Internet: shopping	.15	.03	4.44	.00					.23	.03	7.42	.00
Internet: health	.02	.03	.63	.53	.21	.03	7.33	.00				
												
R-squared	.42				.36				.30			

### Changes in Depression and Health

Table 3 summarizes the lagged dependent variable regression analysis of CES-D depression at a later time from demographic variables and the types of Internet use at an earlier time, controlling for depression at the earlier time. The intercept in this model is the expected level of depression on the second and third questionnaires among nonwhite, unmarried females who did not have a serious illness themselves nor were they caring for someone who did, and who had average levels of education, income, health, depression, and Internet use at the first questionnaire. Those who were more depressed at the initial period were also more depressed at the subsequent period (*P* < .001). Being younger, wealthier, and male were all associated with reductions in symptoms of depression after accounting for initial depression. Using the Internet to obtain health resources was associated with increased depression after accounting for initial depression (*P* = .002). People who used the Internet for communication with friends and family, on the other hand, reported reduced depression after accounting for initial depression (*P* = .002). The changes in depression associated with Internet use were small [69]. Increasing use of the Internet for health purposes by one unit (eg, from 3 to 5 days per week to once a day) was associated with 11% of a standard deviation increase in depression. Conversely, a unit increase in Internet use to communicate with friends and family was associated with 7% of a standard deviation decrease in depression. Using the Internet for meeting new people, escape, entertainment, or shopping was not associated with changes in depression.

**Table 3:** Predicting later depression from prior uses of the Internet, controlling for demographics and earlier levels of depression

	Predicting CES-Depression			
Independent variables	Beta	SE	*t*	*P*
Intercept	1.767	.049	36.36	.000
Male (0=female; 1=male)	-.069	.028	-2.43	.015
Age	-.037	.016	-2.32	.020
White (0=minority; 1=white)	-.031	.048	-.66	.511
Married (0=not married; 1=married)	.007	.031	.22	.825
Education	-.024	.016	-1.46	.143
Income	-.039	.017	-2.37	.018
Depression (time t-1)	.214	.014	15.67	.000
Internet: friends & family	-.041	.015	-2.69	.007
Internet: meet people	.009	.015	.60	.548
Internet: information	.005	.018	.26	.794
Internet: entertainment/escape	.017	.017	1.02	.306
Internet: shopping	.006	.018	.35	.727
Internet: health	.059	.02-	3.23	.002
				
R-squared	.387			

Table 4 summarizes the regression analysis predicting general health at the third questionnaire from demographic variables and the different components of Internet use at the first questionnaire, controlling for prior general health. Being younger, wealthier, and Caucasian were all associated with reporting better health, after statistically accounting for initial health. We found no main effects of Internet use predicting general health at time 3.

**Table 4:** Predicting general health at Time 3 from uses of the Internet at Time 1, controlling for demographic variables and prior health

	Predicting General Health			
Independent variables	Beta	SE	*t*	*P*
Intercept	3.702	.081	45.940	.000
Male (0=female; 1=male)	.010	.045	.220	.829
Age	-.079	.026	-3.020	.003
White (0=minority; 1=white)	.160	.079	2.030	.045
Married (0=not married; 1=married)	-.069	.051	-1.340	.181
Education	.046	.026	1.760	.080
Income	.091	.025	3.570	.000
General health (time 1)	.660	.021	32.160	.000
Internet: friends & family	.019	.024	.810	.418
Internet: meet people	-.029	.028	-1.060	.294
Internet: information	.006	.029	.190	.850
Internet: entertainment/escape	.007	.029	.230	.818
Internet: shopping	.003	.030	.100	.920
Internet: health	-.028	.029	-.970	.336
				
R-squared	.439			

We anticipated that respondents’ initial health and caregiver status would moderate the association between Internet use and subsequent depression and health. We tested this expectation by adding to the models presented in Tables 3 and 4 interactions of the six types of Internet use with the dummy variables representing whether the respondent had a serious illness or took care of a household member with a serious illness at the earlier time period. None of the interactions of Internet use with prior illness or caregiver status was significant (all *P*s > .20; see Multimedia Appendix 2 and Multimedia Appendix 3 for complete results). Thus, these interaction analyses provided no evidence that the impact of using the Internet for health purposes influenced well-being in different ways for those who had a serious illness or who cared for someone with a serious illness compared to a healthy or noncaretaking sample.

## Discussion

The goals of this study were to determine whether people’s use of the Internet to obtain health resources would have consequences for their psychological well-being and physical health, and whether these consequences were comparable to consequences of other uses of the Internet. We examined whether the impact of using the Internet to obtain health resources might be moderated by participants’ initial health or caregiver status. We found that using the Internet to obtain health information was associated with increased depression over approximately 6 to 8 months, while using it to communicate with friends and family was associated with decreased depression. Interestingly, these associations did not depend on the initial health status of the participants (eg, the presence of serious illness) or whether they were the primary caregiver for an ill person. Furthermore, these uses of the Internet were not associated with changes in individuals’ ratings of their general health.

We did not expect that using the Internet for health purposes would be associated with increases in depression. There are, however, several plausible explanations for this finding. First, the Internet has both good and poor quality medical advice [70] that is difficult to for an untrained observer to distinguish [10]. For example, a previous study revealed that only 20% of websites provided correct information on how to take a child’s temperature [71]. Furthermore, only one third of users verify Internet information with their doctor to ensure accuracy [72]. It may be that one source of the increase in depression is the misinformation people get from factually incorrect websites. This may lead to inaccurate self-diagnosis, poor health behaviors (eg, herbal remedies), or potentially unnecessary worry (for both healthy and ill populations). This negative rumination could occur when researching one’s own medical problems or those of loved ones, easily leading to depressive symptoms at a later point in time.

Another possible source of depression may come about when people use online health support groups. While health support groups moderated by doctors, nurses, or trained moderators may be an important source of health information, many online support groups do not have professional moderation, and most are composed of strangers. Both the information and the empathy and other types of emotional support people receive from strangers they meet in online support groups may be less valuable than the resources they could get from offline interactions with family and friends. The advice offered in support groups often consists of personal anecdotes that may not be as helpful as medically relevant information [73]. Moreover, too much time spent in online support groups may displace in-person support and as a result harm the psychological well-being people derive from interaction with friends and family offline [74]. Results from the current study showing that communicating online was associated with declines in depression when the communication was with friends and family but not with new people; and results from prior research [43] showing that communication with strangers online may lead to increased depression suggest that discussion of health problems with strangers online may be problematic.

An alternative interpretation is that people who choose to seek out Internet health resources may be especially sensitive to hypochondriasis or excessive worry about minor health symptoms or perceived health risks. The association between baseline depression and seeking health information online is evidence of this. Internet websites and support groups might be compelling for such persons, as they are rife with lists of symptoms, narratives of pain and grief, dire warnings about treatment side effects, and even graphic photos of diseased organs. Reading about symptoms and anecdotes from patients may cause this group to imagine being ill and to inflate their perceptions of risk. Information and discussions of health problems also may cause them to ruminate [28,29] and increase their anxiety [27,30]. Consistent with this argument is evidence suggesting that those with psychosomatic illnesses are particularly likely to use Internet health resources [23,75], and that people with high levels of health anxiety or hypochondriasis use health resources significantly more than their nonanxious counterparts.

The finding that online communication with friends and family reduced the frequency of depression symptoms is consistent with a large literature on social support [76,77] and warrants little further discussion. If online communication with friends and family increases perceived social support, this could lead to lower depression and improved psychological well-being. Similarly, maintaining contact with friends and family may enhance the quality of relationships, decrease loneliness and social isolation, and improve the nature of the social network, all of which have been tied to lower depression and improved psychological well-being [78-81].

Although one might be concerned about regression toward the mean, this statistical artifact cannot account for results showing that use of the Internet for health information was associated with increases in depression, but use for communication with friends and family was associated with decreases. Indeed regression artifacts would have produced a pattern of results opposite to the ones reported here. Regression toward the mean occurs because of measurement error, when error causes extreme scores at one measurement period to be less extreme at a different period (eg, people who reported many symptoms of depression at the first survey should report fewer at subsequent periods). Models 1A and 1F in Tables 1 and 2 show that people who were initially more depressed were more likely to use the Internet for health purposes and less likely to use it to communicate with friends and family. If their initial depression caused this pattern of Internet use, then regression toward the mean would cause those people who were initially severely depressed (and therefore selected to use the Internet for health purposes) to appear less depressed at the later period.

The absence of significant effects of the Internet on changes in participants’ general health may stem from insensitivity of the health measure. First, we used a single-item measure of general health, which limits our assessment to perceptions of general health as opposed to any one specific symptom. It is also possible that there is no link between physical health and Internet health resource use, or that it is weakened by other behaviors. For example, if using the Internet for health purposes resulted in appropriate treatment seeking, this might mask a possible connection. Unfortunately, since we do not have data on actions taken or health center visits, we cannot determine if this was the case. Thus far, however, the literature does not show such effects, at least not for unevaluated health resources and unsupervised use [82]. Most previous studies of online health resources have measured attitudes rather than behavior change. The few studies examining behavior change [83] have been clinical trials involving physician-supervised, closed Internet sites or moderated support groups rather than free access to Internet resources.

### Limitations

The longitudinal data and analyses reported here allow stronger causal claims about the relationship between Internet use and depression than do cross-sectional data. Because the same individuals were measured at multiple time points, stable characteristics such as demographic differences and personality are automatically controlled when assessing changes in depression. In addition, the lagged dependent variable analysis controls for initial levels of depression and health when predicting subsequent levels of depression and health. Despite the benefits of longitudinal analyses, however, we cannot establish causality solely based on them. In particular, longitudinal analyses do not control for unmeasured variables, such as hypochondriasis, stress, or health behaviors that may also change over time and predict both changes in depression and changes in use of the Internet for health resources. Clinical trials with random assignment are needed to make stronger causal claims.

Our ability to generalize to the US population is limited because we over-sampled Internet users and because only 35% of those initially contacted by telephone completed an outcome measure at least two times. Finally, our data were collected during 2000 to 2002, and the Internet and access to it have changed dramatically since then. The quality of health information and support online may have improved, and Internet users today may no longer use Internet resources in the same fashion as they did during the time period of our study, suggesting the need for follow-up research in this area.

### Conclusion

This study examined the consequences of using Internet health-related resources in a way that other studies have not. We used a national sample including both healthy and ill people, and administered a longitudinal survey to discover if using the Internet to obtain health information or interpersonal communication with friends and family predicted subsequent changes in participants’ reported depression and general health. Our results suggest that using the Internet to obtain health resources is associated with increases in symptoms of depression. This finding cannot be interpreted as a broad effect of being online, since we also showed that communicating online with friends and family was associated with declines in symptoms of depression. However, since we did not control the uses of the Internet chosen by respondents, we cannot determine whether these effects were due to characteristics of the individuals or the nature of the online resources they used, or both. Additional research is needed to determine what leads individuals to seek out health resources online, whether the information that they discover (and believe) is factually correct, and what actions ensue.

## Multimedia Appendix 1

Means/percentages and correlations among variables

## Multimedia Appendix 2

Intervention Characteristics for Interventions Included in the Meta-Analysis

## Multimedia Appendix 3

Predicting health from respondents’ prior use of the Internet and interactions with health and caregiver status
