# Supplementary material for: Effects of Internet Use on Health and Depression: A Longitudinal Study
Source: J Med Internet Res. 2010 Mar 12;12(1):e6. doi: 10.2196/jmir.1149 (PMC3234167; doi:10.2196/jmir.1149)
Supplement: Supplementary file 1 [file jmir_v12i1e6_app1.pdf]

Multimedia Appendix 1: [Means/percentages and correlations among variables]

	Mean	Std	Male	Age	White	Married	Education	Income	Internet: Friends	Internet: Meet	Internet: Information	Internet: Escape	Internet: Shopping	Internet: Health	Caretaker status	Has illness	Health (T1)	Depression (T1)	Health (T2)	Depression (T2&3)
Male (0=female; 1=male)	.41	.49	1.00																	
Age	44.95	17.18	.03	1.00																
White (0=minority; 1=white)	.90	.30	.01	.09	1.00															
Married (0=not married; 1=married)	.61	.49	.06	.25	.10	1.00														
Education	4.67	1.79	-.01	.21	-.02	.19	1.00													
Income	5.12	2.38	.06	.02	.05	.39	.41	1.00												
Internet: Friends & Family	2.86	1.66	-.04	-.28	-.04	-.09	.30	.30	1.00											
Internet: Meet People	1.33	.88	.05	-.28	-.07	-.23	-.02	-.01	.40	1										
Internet: Information	2.52	1.42	.14	-.32	-.06	-.03	.31	.31	.67	.41	1.00									
Internet: Entertainment/Escape	2.38	1.53	.07	-.39	-.13	-.18	.03	.11	.58	.49	.60	1.00								
Internet: Shopping	1.95	.93	.11	-.18	-.05	.05	.35	.32	.57	.27	.69	.55	1.00							
Internet: Health	1.44	.68	-.04	-.09	-.06	-.02	.19	.11	.43	.37	.49	.41	.52	1.00						
Caretaker status (0=no; 1=yes)	.17	.37	-.06	.11	.05	.08	-.05	-.05	-.02	-.05	-.09	-.01	.01	.06	1.00					
Has serious illness (0=no; 1=yes)	.20	.40	.07	.20	-.02	.00	-.11	-.16	-.15	-.01	-.16	-.07	-.07	.03	.21	1.00				
General health (time 1)	3.85	.96	.09	-.21	.01	.04	.17	.27	.20	-.01	.22	.09	.17	.01	-.19	-.43	1.00			
Depression (time 1)	1.70	.52	-.09	-.14	-.01	-.13	-.13	-.16	.00	.12	.02	.19	.00	.09	.10	.23	-.36	1.00		
General health (times 3)	3.82	1.01	.06	-.20	.04	.02	.18	.27	.22	.02	.22	.12	.19	.03	-.13	-.39	.69	-.29	1.00	
Depression (times 2 & 3)	1.73	.55	-.13	-.15	.00	-.10	-.11	-.14	.02	.20	.07	.18	.05	.21	.05	.18	-.31	.59	-.35	1.00

Note: All statistics are based on the first questionnaire, except for for the outcome measures. The depression outcome was measured at times 2 and 3, while the general health outcome was only measured at time 3. Except for means and standard deviations, all entries are Pearson product moment correlations.
