# Supplementary material for: Effects of Internet Use on Health and Depression: A Longitudinal Study
Source: J Med Internet Res. 2010 Mar 12;12(1):e6. doi: 10.2196/jmir.1149 (PMC3234167; doi:10.2196/jmir.1149)
Supplement: Supplementary file 2 [file jmir_v12i1e6_app2.pdf]

Multimedia Appendix 2 [Predicting depression from respondents' prior use of the Internet and interactions with health and caregiver status]

	Predicting depression															
	Main effects				Interactions with general health				Interactions with having serious illness				Interactions with caregive status			
Independent Variables	Coef.	StdErr	t	P>|t|	Coef.	StdErr	t	P>|t|	Coef.	StdErr	t	P>|t|	Coef.	StdErr	t	P>|t|
Intercept	1.767	.049	36.360	.000	1.777	.048	36.640	.000	1.774	.049	36.110	.000	1.763	.049	36.290	.000
Male (0=female; 1=male)	-.069	.028	-2.430	.015	-.063	.028	-2.240	.025	-.069	.029	-2.410	.016	-.065	.029	-2.280	.022
Age	-.037	.016	-2.320	.020	-.053	.016	-3.320	.001	-.040	.016	-2.460	.014	-.043	.016	-2.690	.007
White (0=minority; 1=white)	-.031	.048	-.660	.511	-.035	.048	-.730	.465	-.035	.048	-.740	.461	-.028	.048	-.590	.558
Married (0=not married; 1=married)	.007	.031	.220	.825	.002	.031	.080	.939	.005	.031	.180	.861	.003	.031	.080	.934
Education	-.024	.016	-1.460	.143	-.017	.016	-1.020	.310	-.024	.016	-1.460	.144	-.021	.016	-1.280	.202
Income	-.039	.017	-2.370	.018	-.027	.017	-1.630	.104	-.039	.017	-2.340	.020	-.041	.017	-2.430	.015
Depression (time 1)	.214	.014	15.670	.000	.198	.014	14.030	.000	.212	.014	15.010	.000	.214	.014	15.590	.000
Internet: Friends & Family	-.041	.015	-2.690	.007	-.037	.015	-2.400	.017	-.040	.015	-2.650	.008	-.038	.016	-2.440	.015
Internet: Meet People	.009	.015	.600	.548	.011	.016	.720	.475	.009	.015	.570	.572	.008	.015	.550	.582
Internet: Information	.005	.018	.260	.794	.008	.018	.440	.658	.009	.018	.490	.621	.006	.018	.340	.734
Internet: Entertainment/Escape	.017	.017	1.020	.306	.011	.017	.620	.535	.015	.017	.900	.370	.014	.017	.830	.407
Internet: Shopping	.006	.018	.350	.727	.006	.018	.340	.735	.005	.019	.300	.768	.005	.018	.270	.785
Internet: Health	.059	.018	3.230	.002	.055	.018	3.100	.002	.058	.018	3.160	.002	.055	.018	3.030	.003
General health (time 1)					-.080	.015	-5.360	.000								
Health X Friends & Family					-.031	.018	-1.720	.088								
Health X Meet People					-.005	.020	-.260	.795								
Health X Information					.006	.021	.280	.777								
Health X Entertainment/Escape					.015	.018	.800	.425								
Health X Shopping					.003	.018	.190	.853								
Health X Health					.020	.018	1.090	.279								
Have illness									.023	.014	1.630	.104				
Illness X Friends & Family									.000	.017	.020	.984				
Illness X Meet People									.018	.016	1.100	.271				
Illness X Information									.013	.019	.690	.489				
Illness X Entertainment/Escape									-.001	.019	-.060	.949				
Illness X Shopping									.004	.017	.220	.830				
Illness X Health									-.014	.017	-.850	.399				
Is a cargiver													.029	.013	2.280	.023
Caregiver X Friends & Family													-.013	.014	-.900	.369
Caregiver X Meet People													-.019	.017	-1.150	.254
Caregiver X Information													.014	.019	.750	.455
Caregiver X Entertainment/Escape													.034	.019	1.810	.073
Caregiver X Shopping													-.001	.017	-.070	.942
Caregiver X Health													-.002	.018	-.090	.930
